# Testing a proof of concept model for group couples counseling in family planning in Northern Uganda

**DOI:** 10.11604/pamj.2018.30.179.12670

**Published:** 2018-06-27

**Authors:** Lillian Ojanduru, Dickens Ojamuge, Lauren DuComb, Jeannette Cachan, Esther Spindler

**Affiliations:** 1 Institute for Reproductive Health (IRH), Georgetown University, Washington DC, USA; 2 Save the Children International, Gulu, Uganda

**Keywords:** Family planning, fertility awareness methods, Standard Days Method, Lactational Amenorrhea Method, TwoDay Method, couples counseling, group counseling, proof of concept, Uganda

## Abstract

**Introduction:**

The post-conflict Acholi sub-region of Uganda is undergoing a period of transition that is influencing access, acceptability and use of family planning (FP). Low FP use and high unmet need for FP in Uganda's northern region provides a unique opportunity to test a community-based group counseling approach to reduce unintended pregnancies among young couples. We share findings from a proof of concept testing model in delivering fertility awareness methods (FAM) to groups of couples by trained non-health community youth agents.

**Methods:**

The group counseling model was developed for couples interested in two FAM-standard days method (SDM) and TwoDay Method-within rural communities in Northern Uganda. WALAN was tested in a three-month proof of concept phase, employing 24 direct observations of group counseling sessions; quantitative interviews with 9 couples using either SDM or TwoDay Method; 2 focus group discussions with youth facilitators, and; 9 key informant interviews with providers and leaders.

**Results:**

The proof of concept results suggest model feasibility and acceptability among participating communities. Couples learned how to use FAM correctly. All 9 interviewed female users reported 100% correct knowledge of method use. Couples also reported high levels of satisfaction for both methods. SDM and TwoDay Method participants reported comfort and satisfaction in learning about FAM in small groups with other couples.

**Conclusion:**

The proof of concept phase confirmed intervention feasibility, albeit with some model adjustments. The results were used to inform the pilot intervention, launched in April 2016 within 15 other villages in the same region.

## Introduction

To date, people in the post-conflict Acholi sub-region of Uganda are striving to rebuild their livelihoods and preserve the traditional familial and social structures that were eroded during 20 years of internal conflict. In this low resource setting, low contraceptive prevalence (24%) and high unmet need (34%) [1] are a result of many barriers women and men face in accessing and using family planning (FP). Unplanned births are highest in Northern Uganda, where 54% of all births are unplanned, compared to the national average of 43% [2]. The gap between women's desired and actual births suggests that women in the Acholi sub-region are unable to achieve their fertility goals, posing serious limitations to plans for improving lives of the Acholi people. Providing FP through group counseling can be effective in increasing FP use and acceptability [3]. Growing evidence shows that trained community members can effectively bridge access barriers by providing FP methods directly in the community [4]. Yet, few studies have tested the feasibility of group couples counseling approaches on FP outside health service delivery. Building off this evidence, a proof of concept phase was conducted to test Wake ki Lago Nywal (WALAN) “Be proud with family planning,” a group learning and counseling intervention that relies on non-health youth facilitators, to expand access to FP methods.

**Background on FAM**: As part of WALAN, two FAM developed by the Institute for Reproductive Health, Georgetown University (IRH)-Standard Days Method^®^ (SDM) and TwoDay Method^®^-were offered through a community-based group learning and counseling model to couples in districts of the Acholi region in Northern Uganda. As described in Table 1 below, both SDM and TwoDay Method were developed by IRH to respond to the need for simple, accurate ways for women to identify their fertile window and avoid unprotected intercourse to prevent pregnancy. Given their ease of use and lack of side effects, SDM and TwoDay Method may appeal particularly to couples who currently are not using any method, are relying on some form of “rhythm” or withdrawal, or are dissatisfied with their current method. In addition, since SDM and TwoDay Method are knowledge-based methods, they do not need to be provided by a facility-based health provider. Rather, both methods can be offered directly to consumers through community-based approaches. FAM-including SDM, TwoDay Method, and Lactational amenorrhea method (LAM)-are traditionally offered in a one-on-one provider-client interaction similar to other FP methods. Research suggests that FAM can successfully be taught in groups, but it has seldom been tried outside of small-scale service delivery initiatives [3]. Furthermore, there is robust evidence that FAM can be offered successfully by low-literacy community-level workers (inside and outside of the health system), and appropriate materials have already been developed and tested for these settings [4].

**Table 1:** Fertility awareness methods

FERTILITY AWARENESS METHODS (FAM)	Standard Days Method^®^ (SDM) identifies a fixed fertile window in the menstrual cycle when pregnancy is most likely and is typically used with CycleBeads^®^, a visual tool that helps women track their cycle to know when they are fertile. Results of an efficacy trial showed SDM to be more than 95% effective with correct use and 88% effective with typical use, for women with cycles lasting between 26-32 days.
	TwoDay Method^®^ relies on cervical secretions as the fertility indicator. Results of the efficacy trial published in 2004 showed it to be 96% effective with correct use and 86% effective with typical use TwoDay Method is appropriate for women whose secretions are healthy and who are able to avoid intercourse on days identified as fertile.
	Lactational Amenorrhea Method (LAM) is based on post-partum infecundity and is highly effective if three specific criteria are met: breastfeeding only, no menses, and the baby is less than six months. LAM is more than 99% effective with correct use and 98% effective with typical use

Sources: Arevalo, Jennings, Sinai 2002; Arevalo, Jennings, Nikula, Sinai 2004; Labbock, et al. 1997

**Intervention description**: WALAN was designed as a community-based solution, offering both SDM and TwoDay Method in group counseling sessions. WALAN is implemented in partnership with Save the Children (SC) and the District Community Development Office, through the youth initiative for employment and sustainable livelihoods development (YIELD) project, an agricultural, vocational and apprenticeship training program. Through this platform, WALAN trains youth group members who participate in YIELD to become WALAN youth facilitators. In each implementing community, a pair of WALAN youth facilitators conducts a series of educational community learning sessions on human fertility, healthy timing and spacing of pregnancy (HTSP), LAM and FP. The community learning sessions have the dual purpose of providing fertility and HTSP information to the community at large, while also promoting FP services both within health facilities and through group counseling in SDM and TwoDay Method by the youth facilitators. During the community learning sessions on LAM and FP, participants are either invited to participate in a group counseling session on SDM or TwoDay Method, or are referred for other FP methods at the nearest health center, depending on their preference. Subsequently, group counseling sessions are offered by the youth facilitator pair to small groups of 4-5 couples who desire counseling in their FAM of choice, either SDM or TwoDay Method. After attending the group counseling session, couples are then encouraged to participate in follow-up method support sessions in which they discuss their experiences using the method, review method use, seek advice from their peer couples on potential challenges and encourage one another to use the method correctly. Finally, sensitization meetings are conducted with local community, cultural and religious leaders to ensure support for WALAN activities. Sensitization meetings are also conducted with health providers and Village Health Teams (VHTs) to strengthen linkages between WALAN community activities and referrals for FP services.

## Methods

A small-scale three-month proof of concept phase was tested from October 2015 to January 2016 to understand the delivery and acceptability of the community learning and group counseling model. For this phase, eight pairs of facilitators were trained on human fertility, FP/HTSP and FAM as well as co-facilitation skills to deliver community learning and group counseling activities across eight villages in the districts of Gulu and Nwoya. The purpose of the proof of concept testing phase was to determine (a) whether to continue moving the intervention forward into the next pilot phase and if so, (b) what changes should be made to increase the potential scalability and desired effect of the intervention. The testing phase aimed to answer the following three assessment questions: can trained community-based youth facilitators implement the WALAN community learning and group counseling session activities? Can couples learning about FAM through a group counseling session in their community gain the knowledge to use SDM and TwoDay Method correctly? What are community stakeholders? perceptions and acceptability of WALAN? A mixed methods design was used to answer the above questions, triangulating data from WALAN youth facilitators, FAM users and community key informants. Observational monitoring data were collected, including attendance records, session observations and facilitator debriefs. The team collected a total of 34 FAM user interviews (SDM and TwoDay Method) with 9 female users and 9 male partners at two time points (with two losses to follow-up), two focus group discussions (FGDs) with 16 youth facilitators, and nine key informant interviews (KIIs) with health service providers and community leaders. FAM user interviews with the 9 couples were collected approximately three weeks after participants attended their first group counseling session and again at a second follow-up point, six to eight weeks post-group counseling session. Female and male partners were interviewed separately; for every couple, the female user was interviewed first and then her permission was asked to interview the male partner.

## Results

Results from interviews with FAM users, key informants and youth facilitators, in addition to session observations and facilitator debriefs illustrated four main findings about intervention reach, facilitator competence, couples' knowledge of method use and community acceptability. Each individual finding supports the feasibility of the group counseling model, although appropriate adjustments were made to the model as a result of these findings.

**Figure 1:**
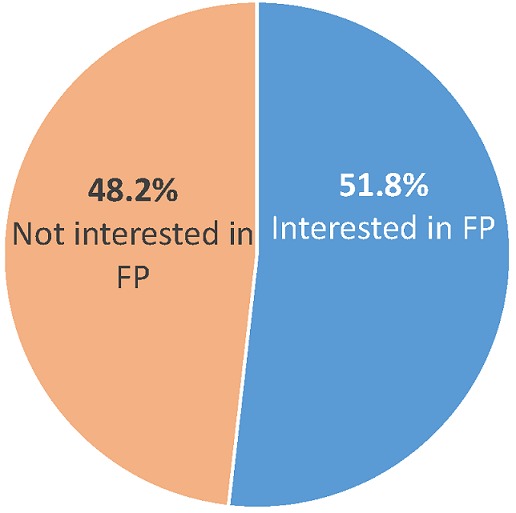
Community learning participants interested in FP methods (n = 498)

**Figure 2:**
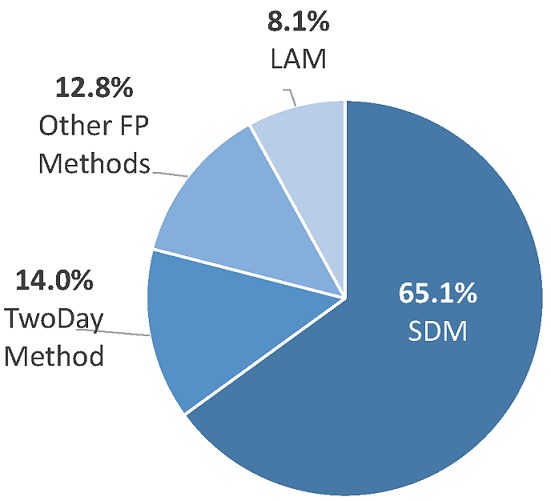
Community member interest in FAM and other FP methods, by type (n = 258)

**Intervention reach**: The proof of concept results show high demand for and interest in community learning and group counseling activities. In the period of three months, a total of 32 community learning sessions took place on fertility, HTSP and FP, and LAM across eight villages to approximately 498 participants. Of these, approximately 63% were female and 37% male; and about 61% to 76% had children. The majority of participants were young, with 51% of participants being 20-25 years old, 20% 15 to 19 years and 26% between 26 to 35 years of age. Only 2% were above 35 years. Below, Figure 1 and Figure 2 show that just over half of the 498 community learning participants (51.8%) were interested in either FAM or another FP method. Of these 258 participants, approximately 168 were interested in SDM, followed by 36 for TwoDay Method, 33 for other FP methods provided at the health center and 21 were interested in LAM. Community learning sessions were used as informative sessions on fertility and FP topics, as a mobilization venue for promoting FP, including group counseling sessions on FAM, and a way to provide linkages to other FP methods at the health center. Over the three month testing phase, a total of 38 group counseling sessions-including 25 SDM and 13 TwoDay Method sessions-were delivered to approximately 211 participants across the eight villages (average ~5.6 couples per session). However, some of these participants were returning couples who went back to method support sessions after the first group counseling session. Adjusting for couples who returned to more than one group counseling session, approximately 10 couples participated in group counseling in each site, totaling 80 couples who attended a group counseling session and received either SDM or TwoDay Method. The proof of concept intervention also explored options for referring community members to FP services, including the testing of a FP invitation card to seek more FP information at nearby health facilities.

**Facilitator competence**: During facilitators' activities in the community, external observers accompanied the pair of facilitators and applied a competency checklist to assess the delivery of community learning, group counseling and method support sessions. The competency checklist collected information on: counseling principles, facilitation skills, screening couples, teaching the method and supporting the couple to use the method. During SDM and TwoDay Method group counseling sessions, facilitators were ranked on the following categories: 1) Correct use of job aids; 2) Correct explanation of method; 3) Client screening; 4) Safe learning environment and; 5) Demonstration of essential skills. Each of the five categories was ranked as either, “Did well;” “Did partially;” or “Did not do at all.” A total of five SDM facilitator pairs and three TwoDay Method pairs were observed. In general, youth facilitators performed best in their use of job aids and ensuring a safe environment. Similar to the community learning sessions, both SDM and TwoDay Method facilitators almost unanimously scored perfectly on using their job aids and ensuring a safe counseling environment. One possible explanation could be that facilitators strengthened their skills in both areas due to their conducting community learning sessions the month prior to starting the group counseling sessions. Five of the six observed facilitators completed the screening for SDM eligibility correctly, covering both the cycle length as well as the couple agreement criterion. For TwoDay Method, however, only one of the three facilitators observed mentioned who is eligible to use the method. In addition, difficulties recruiting eligible users for the FAM user interviews below suggest that facilitators may need to improve screening. For instance, some participants who were contacted to participate in FAM user interviews had received the method but were either pregnant prior to attending the session or were post-partum and therefore were unable to use the method.

**Both SDM and TwoDay Method facilitators can explain the methods correctly**: Both groups of SDM and TwoDay Method facilitators observed were able to cover the essentials of each method's daily use, with five out of the six SDM facilitators explaining how Cycle-beads work, including monitoring for short and long cycles. TwoDay Method facilitators similarly covered the essentials of secretions and how to identify the fertile days, with all three facilitators explaining these key points (2 explained fully and 1 partially).

**Couples' knowledge of method use**: Correct knowledge of method use was assessed by interviewing five SDM and four TwoDay Method couples at two time points. All five SDM female participants scored 100% correct knowledge of method use both at the first interview and again three weeks later at the second data collection point (Table 2). Only three of the five male partners demonstrated 100% correct knowledge of method use at the first data collection point. However, all of the men improved by the second data collection point, each scoring 100% correct knowledge of method use. Potential reasons for men's knowledge improvement include communication with their partner about the method use and their return to more method support sessions to learn more about the method. In contrast, all four of the TwoDay Method couples interviewed, including female users and male partners, showed correct knowledge of method use when prompted to show use of their TwoDay Method calendar at both time points (Table 3). The second key criterion for using either SDM or TwoDay Method is the ability to either abstain or use condoms during fertile days. The assessment findings confirm that the five SDM couples and four TwoDay Method couples used a combination of both abstinence and condoms to avoid pregnancy on fertile days. SDM and TwoDay Method users managed fertile days in different ways. In particular, SDM participants reported using condoms more frequently than TwoDay Method participants, while TwoDay Method more commonly reported use of abstinence during fertile days.

**Table 2:** Correct knowledge of method use, SDM Users (n = 5 couples)

	FEMALE USERS		MALE PARTNERS	
Measures	1^ST^ Interview (n=5)	2^nd^Interview (n=4)	1^st^Interview (n=5)	2^nd^Interview (n=5)
1. Move ring to red bead	5	4	4	5
2. Move ring to next bead daily	5	4	5	5
3. Use condoms or abstain when ring on white bead	5	4	5	5
4. Brown beads are safe days	5	4	4	5
5. Move ring back to red bead at start of next period	5	4	3	5

(Source: FAM User Interviews)

**Table 3:** Correct knowledge of method use, TwoDay Method Users (n = 4 couples)

	FEMALE USERS		MALE PARTNERS	
Measures	1^st^ interview (n=4)	2^nd^interview (n=4)	1^st^ interview (n=4)	2^nd^ interview (n=4)
1. Marked dots of period on the calendar	4	4	N/A	N/A
2. Marked each day of cycle	4	4	N/A	N/A
3. Correctly identifies fertile days	4	4	4	3
4. Meaning of secretions either today or yesterday	4	4	4	3

(Source: FAM user interviews; N/A = questions not asked to respondent)

**Community acceptability of WALAN**: Of the nine couples sampled during the FAM user interviews, all SDM and TwoDay Method users reported being comfortable discussing and sharing about sensitive topics in small group counseling sessions with other couples. Both male and female SDM and TwoDay Method users were comfortable sharing about the menstrual cycle as well as sharing about issues they may have had with their partner, including abstaining and/or using condoms during fertile days. The proof of concept findings show promising levels of method satisfaction and willingness among some of the FAM users to recommend others that they attend group counseling sessions. Reasons for satisfaction included the ability to manage or “control” the method, lack of side effects and fact that methods had been brought “closer” to the community: *"Because I have learnt so many things about me as a woman and I have also realized that the method is good since it is not painful, doesn't need money, no need to go to hospital. You can monitor your secretions by yourself, so I feel that the method is favorable for us who are deep in the villages."*-Female TwoDay Method User, Anaka Sub County, Nwoya district.

*"You are in control of the bead. It is easy to use and also manage. Since it is a natural method, it can't cause any side effects like other FP methods."* "-Female SDM User, Bungatira Sub County, Gulu District. Despite reported satisfaction with method use, some women using TwoDay Method felt that checking for secretions on a daily basis can sometimes be challenging given their work schedule in the fields. As well, one TwoDay Method female user and another TwoDay Method male partner noted that their community peers discouraged them from using the method, stating that checking secretions using your fingers was considered to be 'not healthy.' This finding was confirmed during FGDs with youth facilitators and key informant interviews with community leaders, whom expressed that TwoDay Method can be considered unhygienic and culturally inappropriate.

Key informant interviews also indicated that health workers, VHTs and community leaders were accepting of the WALAN intervention. However, some of the health workers were concerned about confidentiality issues in a group counseling setting. One VHT from Anaka Subcounty explained that to ensure confidentiality, the model should be flexible enough for facilitators to address individual needs outside of the group setting. One clan leader mentioned the need to spread the “good news” of the WALAN intervention through different communities. However, they also noted some opposition to the intervention in the community. While leaders recognized that WALAN aimed to help “space” births, some in the community saw it as a way of “stopping” births all together. Interviews with health workers, VHTs and local leaders provided insights into the overall perceptions of key community gate-keepers related to FP and the involvement of non-health agents in delivering FP information and FAM counseling, which helped fine-tune intervention messaging and counseling components.

## Discussion

The aim of this proof of concept phase was to inform the development of a larger pilot phase of the intervention, launched in April 2016. The findings confirmed the feasibility of the WALAN intervention, albeit taking into account model adjustments. First, the findings suggest that youth facilitators, despite not being trained health professionals, can deliver group counseling sessions to interested couples for either SDM or TwoDay Method. However, facilitators require more supportive supervision in screening participants and improving facilitation skills. Community leaders and providers reported that the small group counseling model outside of the health system is an acceptable approach. However, both leaders and providers mentioned that this setting may not be appropriate for couples and women who need to disclose very sensitive information. As a result, additional training for youth facilitators has been provided in WALAN's pilot phase to encourage couples and individuals to approach facilitators individually and prepare youth facilitators to address sensitive issues when they are approached. The group counseling model also explored the engagement of men as partners in SDM and TwoDay Method use. The findings show that men are not only interested and engaged, but also satisfied with method use. However, the test findings showed that these men were not a reflection of the entire community, and that some men either did not return to method support sessions, or did not want to participate at all in a group counseling session. The proof of concept phase tested the provision of group counseling sessions to couples only, but some community stakeholders expressed the importance of providing group counseling sessions to women only as well. As a result, in the pilot phase group counseling sessions were offered to both couples and women separately during the pilot phase of the intervention. The counseling model also tested whether couples who learn about SDM and TwoDay Method in a community group setting can use the method correctly. The effectiveness of FAM depends on whether the couple can learn the method correctly, in addition to abstaining or using condoms during fertile days. Results showed that couples learned how to use the methods correctly and managed fertile days using a combination of methods (i.e condoms and abstinence). Results from the proof of concept confirmed high levels of acceptability and willingness of both participants and users to recommend others in their social networks that they attend the community learning and group counseling sessions. Reasons for high acceptability across all FAM users, facilitators and leaders interviewed included the ability to manage or “control” the method, lack of side effects and the fact that access to these methods had been brought “closer” to the community. One key concern mentioned by leaders and service providers included addressing intimate needs and individual questions in a group environment. However, the focus group discussion results suggest that youth facilitators also provided a separate, private space for individuals and/or couples to address them privately about personal issues, outside of the group counseling setting.

## Conclusion

Correct knowledge of SDM and TwoDay Method use among users and facilitators' ability to deliver the sessions indicate that the curriculum and the process in which it was implemented were sufficient to test and move the intervention toward the pilot phase. Findings from the proof of concept phase were used to refine aspects of WALAN, which launched the pilot phase in April 2016. Pilot activities occurred in 15 villages within the Gulu, Nwoya and Amuru districts of Northern Uganda.

### What is known about this topic

- FAM are modern FP methods, scientifically tested and proven effective (SDM is 95% effective with correct use and 88% with typical use; TwoDay Method is 96% effective with correct use and 86% with typical use);
- FAM is widely offered in one-on-one counseling in facility settings by medical professionals as well as in community settings by community health workers;
- In Uganda, FAM are included in the national FP norms and guidelines and offered most often in community settings by local service NGOs.

### What this study adds

- Whereas counseling in FAM is not a new phenomenon, FAM has never been tested in a group counseling session format delivered by non-health community youth agents;
- The group counseling model is also testing a couples counseling approach, bridging the evidence gap on best strategies to engage men in FP counseling and decision-making;
- This study shares a proof of concept methodology and process for testing a new FP counseling model, strengthening the evidence-base around process methodology and evaluations.

## Competing interests

The authors declare no competing interests.
